# Indications and outcome of thoracotomy in a new cardiothoracic unit in sub-Saharan Africa

**DOI:** 10.1186/1749-8090-10-S1-A232

**Published:** 2015-12-16

**Authors:** Abubakar Umar, Salisu Ismail, Ray Bayo, Ukwuani Solomon I, Orakwe Obinna

**Affiliations:** 1 Cardiothoracic Surgery Unit, Department of Surgery, Usmanu Danfodiyo University/Usmanu Danfodiyo University Teaching Hospital, Sokoto, Nigeria; 2 Cardiothoracic Surgery Unit, Department of Surgery, Usmanu Danfodiyo University Teaching Hospital, Sokoto, Nigeria; 3 Federal Medical Center Asaba, Delta state, Nigeria

## Background/Introduction

Thoracotomy is a major thoracic surgery procedure that has various indications and can be marred with various complications and occasionally dismal outcome especially in resource poor environment like ours.

## Aims/Objectives

To determine the demographic pattern, indications, complications and challenges of open thoracotomy in a new cardiothoracic unit in sub-Saharan Africa.

## Method

It is a prospective study of patient admitted into the unit since its inception between January 2012 and May 2015. Patient's demographic data, clinical presentation, indications for and outcome of thoracotomy were analysed.

## Results

A total of 40 patients were admitted and treated during the period with a male to female ratio of 2.6:1. The mean age was 29.5 years. The most common indication for thoracotomy in our series is chronic empyema thoracis, which accounted for 16 (40%) of all the thoracotomies, chest trauma was an indication in 12 (30%) of cases. Eight patients out of 12 (66.7%) had emergency thoracotomy. One patient (2.5%) had thoracotomy and bronchotomy for removal of a long-standing foreign body in the right lower lobe bronchus. Three patients (7.5%) died, 2 died intraoperative and one died in the intensive care unit after emergency thoracotomy following penetrating chest trauma.

## Discussion/Conclusion

Chronic empyema thoracic is currently the most common indication for thoracotomy in our setting. This can be attributed to the fact that we are still battling with various forms of pulmonary and pleural infections that are poorly treated and most of these patients present late to the hospital. The outcome of thoracotomy is good despite the fact that it is a new unit and our resources are limited.

